# From a mouse: systematic analysis reveals limitations of experiments testing interventions in Alzheimer's disease mouse models

**DOI:** 10.1002/ebm2.15

**Published:** 2016-07-22

**Authors:** K.J. Egan, H.M. Vesterinen, V. Beglopoulos, E.S. Sena, M.R. Macleod

**Affiliations:** ^1^ Centre for Clinical Brain Sciences University of Edinburgh Edinburgh UK; ^2^ Centre for Cognitive and Neural Systems University of Edinburgh Edinburgh UK; ^3^ Department of Clinical Neurosciences, Western General Hospital University of Edinburgh Edinburgh UK

**Keywords:** systematic review, Alzheimer's disease, mouse models, preclinical trials

## Abstract

The increasing prevalence of Alzheimer's disease (AD) poses a considerable socio‐economic challenge. Decades of experimental research have not led to the development of effective disease modifying interventions. A deeper understanding of in vivo research might provide insights to inform future in vivo research and clinical trial design. We therefore performed a systematic review and meta‐analysis of interventions tested in transgenic mouse models of AD. We searched electronically for publications testing interventions in transgenic models of AD. We extracted data for outcome, study characteristics and reported study quality and calculated summary estimates of efficacy using random effects meta‐analysis. We identified 427 publications describing 357 interventions in 55 transgenic models, involving 11,118 animals in 838 experiments. Of concern, reported study quality was relatively low; fewer than one in four publications reported the blinded assessment of outcome or random allocation to group and no study reported a sample size calculation. Additionally, there were few data for any individual intervention—only 16 interventions had outcomes described in 5 or more publications. Finally, “trim and fill” analyses suggested one in seven pathological and neurobehavioural experiments remain unpublished. Given these historical weaknesses in the in vivo modelling of AD in transgenic animals and the identified risks of bias, clinical trials that are based on claims of efficacy in animals should only proceed after a detailed critical appraisal of those animal data.

## Introduction

The burden of Alzheimer's disease (AD) is expected to rise substantially in the coming years, and the development of treatments which might halt or reverse disease progression is therefore of considerable importance. Around 5% of AD cases are familial, and the identification of the genetic causes of these has informed the generation of a number of transgenic mouse lines expressing mutations known to cause human disease, which have been used to model aspects of AD. Such models have become increasingly sophisticated and some transgenic lines manifest several features of AD including tau neurofibrillary tangles, amyloid plaques and cognitive change.^1^

One use to which these mice have been put is in efforts to develop treatments for AD, with over 300 interventions having been tested in the Tg2576 mouse model alone.^2^ However, efficacy observed in animal studies has proved to be a poor guide to efficacy in clinical trials, and a deeper understanding of the use and limitations of studies using transgenic mouse models might increase our understanding of the possible reasons for this. Systematic review and meta‐analysis are useful tools to assess the internal and external validity of a research field.^3^, ^4^, ^5^ Systematic review can provide an unbiased ascertainment of publications in a given area while meta‐analysis allows the calculation of global estimates of efficacy and of efficacy in sub‐groups of studies with shared characteristics. Reed et al. have previously reported the use of these techniques on data from their own laboratory to derive more precise estimates of change in performance in the Morris water maze (MWM), and while they did not address issues of internal or external validity they did demonstrate the feasibility of this approach.^6^ In the modelling of other neurological conditions such as focal cerebral ischaemia, multiple sclerosis and Parkinson's disease, the consistent finding has been that publications reporting measures to reduce the risk of bias give consistently lower estimates of efficacy.^3^, ^4^, ^5^ The presumption that these lower estimates of efficacy were more likely to be accurate led to the development of guidelines for good laboratory practice^7^ and informed the development of the ARRIVE statement.^8^

Here, we use a similar approach to ascertain the prevalence and impact of factors known to increase the risk of bias in the literature describing the modelling of AD in animals. We use systematic review to identify relevant publications and a predefined analysis plan to provide a systematic description of the published literature reporting the testing of interventions in transgenic mouse models of AD. From these publications, we extracted data for reported pathological and neurobehavioural outcome measures to assess the impact on reported efficacy of study features which might increase the risk of bias and to make an assessment of the presence and magnitude of any publication bias.

## Methods

We have previously described the CAMARADES AD dataset, including the search strategy used, criteria for inclusion and exclusion, data extraction and the data dictionary for that dataset, which is available in Figshare (http://figshare.com/articles/Interventions_tested_in_preclinical_studies_using_transgenic_mouse_models_of_AD/1185428). The study protocol, including dates of and rationale for revisions to that protocol, is also available (www.camarades.info).

Briefly, we searched Pubmed, EMBASE and ISI Web of knowledge for studies on transgenic animal models of AD using the search terms [“targeted deletion” OR “overexpression” OR “knock out” OR “vector” OR “transgenic”] AND [“dementia” OR “tau” OR “mild cognitive impairment” OR “Alzheimer's disease”] within the limit “animals”. The searches were taken from 1995 (when the first transgenic AD mouse model was described) and were conducted in January 2009.

Two reviewers (KE and MM) independently screened the title and abstract of identified publications. Studies were included if they reported the testing of an intervention in any amyloid, tau or presenilin‐based transgenic mouse model of AD. We excluded studies using mice with additional genetic manipulations (e.g. COX‐2 knockout). We excluded publications without an appropriate control and those using combination therapies. For studies reporting active immunization using amyloid beta peptides, we extracted data on the specific fragments of Aβ used and did not examine adjuvants, modifications or vectors in further detail.

### DATA EXTRACTION

We included studies where outcomes were reported as changes in pathology or neurobehaviour. For pathological outcomes, we included data for plaque burden, cellular infiltration, neurodegeneration, and the abundance of amyloid beta 40, amyloid beta 42 and tau. For plaque burden, we included all related outcomes such as plaque number, plaque density and plaque area. We limited the analysis to plaques identified by immunohistochemistry to reduce the possibility of intra‐staining variation and to minimize aggregation bias attributed to alternative staining techniques. To improve the specificity of plaque burden analyses, we focused only on extracellular plaque burden. Outcomes measuring tau included both overall reductions in tau and reductions in phosphorylation status, and for neurodegeneration, we included both direct measures of neuronal loss (such as cell counting or synapse density) and indirect measures such as caspase 3 activation.

For all neurobehavioural outcomes except the acquisition phase of the MWM, we only included data for the last time point. For the acquisition phase of the MWM, we extracted data for all time points provided that the position of the platform had remained constant. For the probe phase of the MWM, we did not include data for reversal task behaviour and for time in opposite or adjacent quadrants.

To assess factors relating to risk of bias and study quality, we recorded the reporting of (1) random allocation to group, (2) blinded assessment of outcome, (3) sample size calculation, (4) compliance with animal welfare legislation and (5) a statement declaring a possible conflict of interest.

For the purposes of analysis, we grouped transgenic mice into six broad classifications: (1) amyloid precursor protein (APP) mutations (e.g. APP_Swe_ or APP_Swe/Ind_), (2) Presenilin 1 mutations (e.g. PS1_L235P_ or PS1_M146L_), (3) tau mutations (e.g. T44 or tgP301L), (4) APP, PS1 or PS2 mutations, (5) 3xTgAD′ (those with mutations in APP, PS1 and tau (e.g. APP_Swe_/PS1_M146V_/tau_P301L_) and (6) other (AD11, Tg13592 and Nse/ps2m transgenic mice). For active immunization by fragments of amyloid beta, we classed interventions by the fragment of amyloid used only.

We assessed the potential impact of publication bias using Egger regression, funnel plot asymmetry and “trim and fill” techniques. We first derived overall estimates of the impact on pathological and neurobehavioural outcomes. To address the possibility that observed small study effects might be because of systematic differences between types of study (for instance, one outcome measure detecting large effects but with large variance and another showing smaller effects with smaller variance), we conducted sensitivity analyses using subsets of datasets aggregated by outcome measures, transgenic model groups and brain regions (not shown).

### STATISTICS

The details of and the rationale for the statistical approach taken is described in detail elsewhere.^9^ Briefly, we defined a comparison as an outcome measured in a group of treated transgenic animals compared with that in untreated control transgenic animals. For each comparison, we extracted data for numbers per group, mean outcome and variance. Where a single control group served multiple experimental groups, we corrected the weighting given to that group in the meta‐analysis by dividing the number of animals in the control group by the number of treatment groups served. For each comparison, we calculated a standardized mean difference effect size. We aggregated individual effect sizes using DerSimonian and Laird random effects meta‐analysis.^10^

To examine any impact of randomization and blinding on the effect sizes estimated, we first calculated summary estimates of efficacy for each outcome using DerSimmonian and Laird random effects standardized mean difference meta‐analysis. For each of randomization and blinding and for each outcome measure, we calculated efficacy in studies at high and low risk of bias, and then, calculated an effect size for the difference between these. We then pooled these using random effects meta‐analysis to give an estimate of the overall impact of these two potential sources of bias. We then used stratified meta‐analysis to test whether this impact was different for pathological or behavioural outcomes.

All stratified analyses were pre‐specified and a subsample of the dataset (10%) was crosschecked by a blinded reviewer for consistency. Significant differences between n groups were assessed by partitioning heterogeneity using the χ^2^ statistic with n – 1 degrees of freedom. We adjusted our critical significance threshold to account for the number of comparisons using a Holm–Bonferroni correction (p < 0.025 for overall estimates, <0.017 for individual neurobehavioural outcomes and <0.009 for pathological outcomes).

## Results

**Figure 1:**
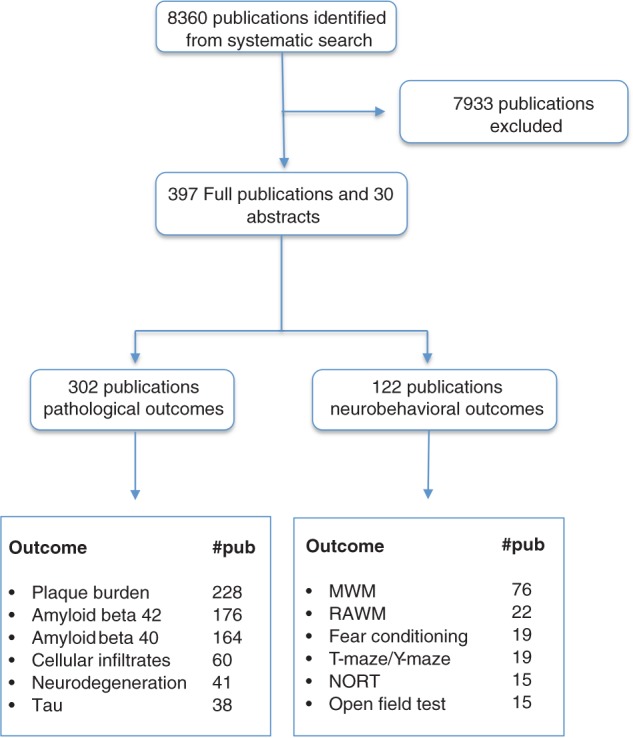
Systematic search process alongside the number of publications reporting specific pathological and behavioural outcomes. From the included 427 publications (397 full publications and 30 abstracts), we identified a number of main outcomes including, plaque burden, amyloid beta 40, amyloid beta 42, tau, neurodegeneration, cell infiltrates and neurobehaviour and the number of publications is shown in the bottom two boxes. As each publication can have more than one outcome, the numbers in the bottom two boxes do not necessarily add up to the numbers above. MWM, Morris water maze; RAWM, radial arm water maze; NORT, novel object recognition test.

**Figure 2:**
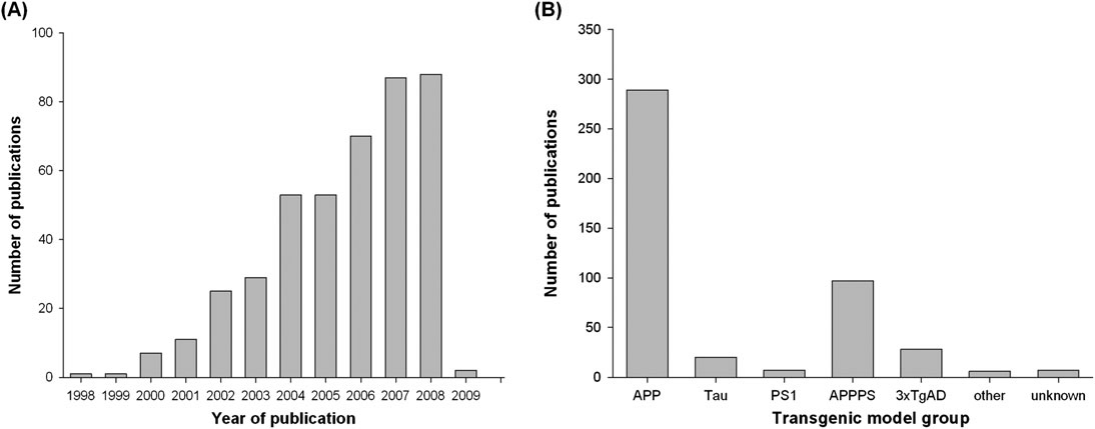
Number of publications by year and frequency of transgenic model groups. Since 1998, there has been an increase in the number of publications per year until the search was conducted (**A**), for transgenic mouse model groups, we categorized transgenic mouse models into the transgenic model group used which included; tau, PS, amyloid precursor protein (APP), 3xTgAD, other and unknown transgenic groups (**B**). See methods for details of these transgenic model groups.

We identified 8,360 publications of which 427 met our inclusion criteria (see Figure 1 for flow diagram and Figure 2A for year by year breakdown). These reported the testing of 357 different treatment strategies in 55 different transgenic models (Table 1). Pathological or neurobehavioural outcomes were reported from 838 experiments involving 11,118 animals. APP transgenic mice were used in 289 of 427 (68%) publications (Figure 2B) including the most commonly reported mouse, the Tg2576 mouse which was used in 149 publications. 8.9% (38/427) of publications reported using more than one transgenic mouse model.

**Table 1:** Transgenic mouse model use: our systematic search identified the description of 55 different transgenic models which were categorized according to transgenic model groups; APP, PS1, tau, APPPS1, 3xTgAD, unknown and other. Numbers represent the number of publications

Transgenic model group	Transgene	Total
3xTgAD	3xTgAD	28
APP	Tg2576	149
	TgCRND8	30
	APPV717F	27
	APPswe	19
	APP751lon/swe	15
	J20 APPSWE/IND	15
	APP23	14
	APPlon	12
	APP	4
	APPlon/swe	4
	APP‐YAC	2
	TG‐SwDI	2
	APP51/16	1
	APP695	1
	APP695lon/swe	1
	APParc (E22G)	1
	APPV717F (APOE KO)	1
	APPV717I	1
	APPV717I‐CT100	1
	CamKII ttA × tet APPswe/ind	1
	tgNORBA	1
APPPS	APPswe/PS1dE9	41
	APPswe/PS1M146L	23
	APPswe/PS1	7
	APPswe/PS1A246E	5
	APPswe/PS1M146V	5
	APPV717I/PS1A246E	4
	APPPS1	3
	APPswe/PS1L166P	2
	APPswe/PS2N141I	2
	APPV717F/PS1M146L	2
	APP23/PS45	1
	APP24	1
	APP695K594N,M595L/PS1de9	1
	APPswe/PS1P246L	1
Other	AD11	4
	Nse/ps2m	1
	Tg13592	1
PS1	PS1	1
	PS1dE9	1
	PS1‐L235P	1
	PS1M146L	2
	PS1M146V	2
tau	tgP301L	6
	T44	4
	p25	2
	tau V337M	2
	GSK‐3/VLW	1
	GSK3betaS9A	1
	NFT P301S/K257T	1
	NSE/APPsw	1
	P301S/K257T	1
	pNSE/htau23	1
Unknown	Unknown	7

Pathological outcomes were reported in 302 publications. Two hundred and twenty‐eight publications reported changes in plaque burden, determined using immunohistochemistry in 378 experiments described in 198 publications. Changes in amyloid beta 40 and amyloid beta 42 were reported in 164 (388 experiments) and 176 (389 experiments) publications, respectively. Changes in tau were reported in 38 publications (84 experiments) and measures of neurodegeneration were reported in 41 publications (64 experiments). Sixty publications (89 experiments) reported changes in the abundance of cellular infiltrates. Overall, pathological outcome was improved by 0.78 SD (95% confidence interval [CI] 0.71–0.85, p < 0.005) although as anticipated there was substantial heterogeneity between studies (χ^2^ = 4,504, I^2^ = 83.9%).

Neurobehavioural outcomes were reported in 122 publications; 76 publications reported outcomes from the MWM of which 130 experiments reported data from the acquisition phase and 113 reported data from the probe phase. Outcomes from the radial arm water maze were reported in 22 publications (41 experiments), from fear conditioning in 19 (45 experiments), from the T or Y maze in 19 (28 experiments) and from the novel object recognition task in 15 (25 experiments). Data from the open field test were reported in 15 publications involving 24 experiments. Performance in wild‐type mice was reported in 18 experiments; in transgenic mice mobility in the open field was higher than wild type in 11 experiments (including 10 of 11 studies using 3xTgAD lines and one using APP lines) and lower than wild type in 7 experiments (including 4 of 7 studies using APPPS lines, 3 of 7 using APPS lines) (Figure S1, Supporting Information). These data were not analysed further. Overall, neurobehavioural outcome was improved by 0.61 SD (95% CI 0.54–0.69, p < 0.005, χ^2^ = 586.2, I^2^ = 56.0).

### METHODOLOGICAL VARIATION

#### Age of animals used and interventions tested

**Figure 3:**
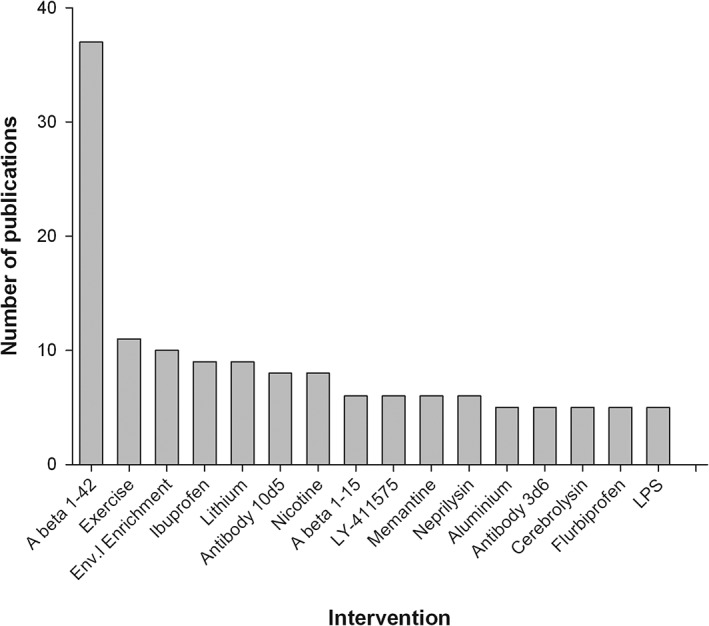
Interventions with outcomes reported in five or more publications. The 16 most commonly tested interventions where outcomes were reported and the number of publications they feature in. A beta 1‐42: active immunization using amyloid beta 1‐42, A beta 1‐15: active immunization using amyloid beta 1‐15, Env. Enrichment, environmental enrichment; LPS, lipopolysaccharide.

Across all outcomes the median age at which treatment was begun was 168 days (IQR[inter‐quartile‐range] 84–311) and the median age at which outcome was assessed was 308 days (IQR 175–420). We identified 357 different interventions, but few were tested extensively, only 16 being reported in five or more publications (Figure 3). We chose not to categorize interventions by drug class or mode of action because we were not confident of any criteria with which we could reliably base such a categorization.

#### Pathological experiments

For all outcome measures, there was considerable variation in the way in which that outcome was measured. For instance, changes in tau were reported in 38 publications representing 84 experiments (involving 984 animals). Changes in both the abundance and the phosphorylation state of tau were reported from 28 experiments, with changes in abundance alone reported in 25 experiments and of phosphorylation alone in 31 experiments. Changes in tau phosphorylation were reported from nine different phosphorylation sites.

#### Neurobehavioural experiments

**Figure 4:**
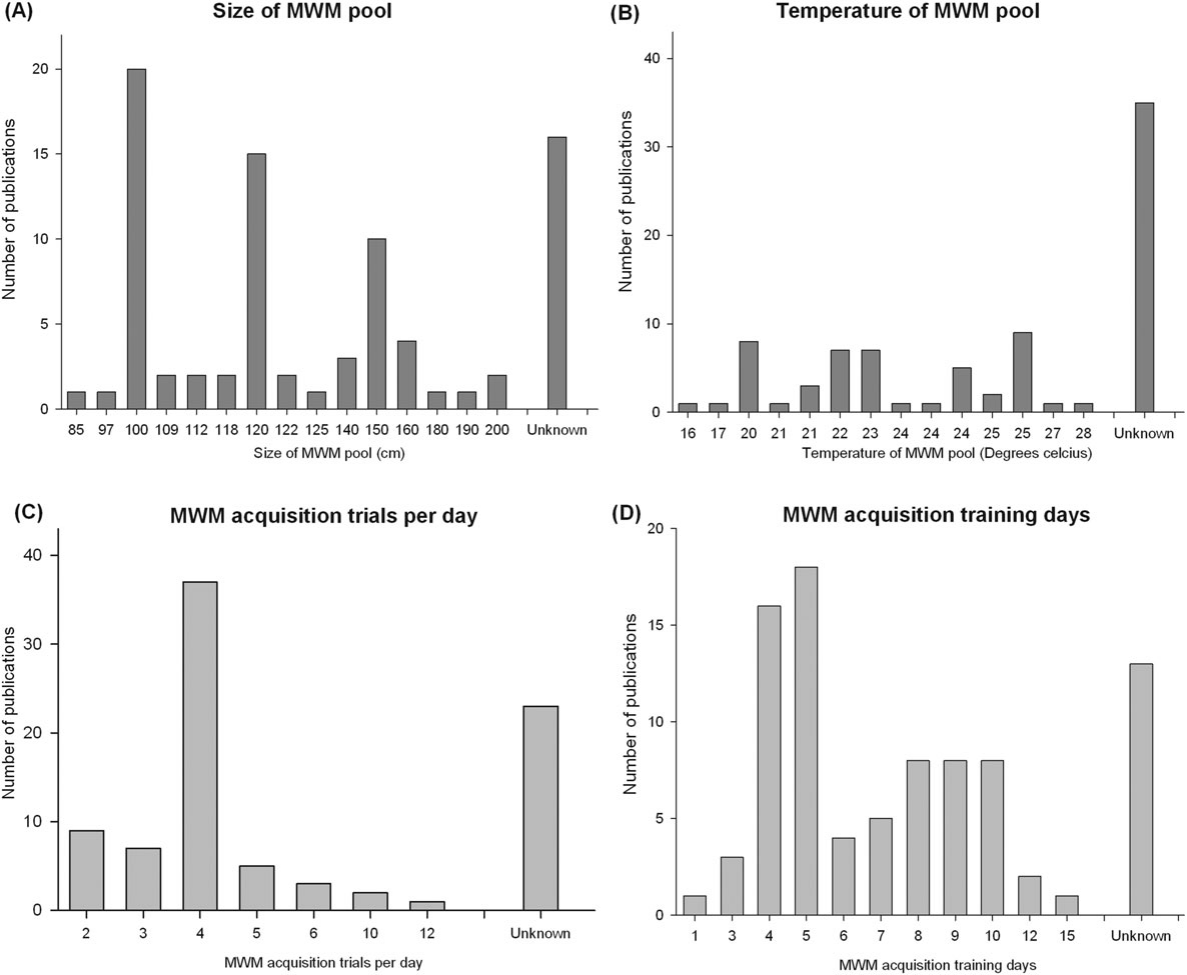
Variation in Morris water maze experimental design. The use of the Morris water maze (MWM) varied considerably with respect the size of the pool used (**A**), water temperature (**B**), the number of training trials per day (**C**) and number of days trained (**D**).

The most commonly used neurobehavioural test was the MWM (83 publications). These reported data from 130 experiments involving 2,151 animals tested during the acquisition phase and 113 experiments involving 2,018 animals tested during the probe phase. Again, there was substantial variation in the details of how these experiments were performed; water temperature ranged from 16°C to 28°C, the size of the pool varied from 85 to 200 cm, the number of trials per day ranged from 2 to 12 and number of training days ranged from 1 to 15. Many publications did not give details of these variables (Figure 4).

From the acquisition phase, seven different outcomes were reported; “latency” (107 experiments), “path length” (57 experiments), “trials to criterion” (2 publications), “search error” (2 experiments) “cumulative distance to platform” (1 experiment) “time in outer zone” (1 experiment) and “difference in path length” (1 experiment). For the probe phase there was if anything greater variability in its use; 12 principal methods were used to measure efficacy, and within the 57 studies which used the probe phase of the MWM there were 59 different approaches used to demonstrate efficacy (see Table S1).

### STUDY QUALITY

Measures associated with risk of bias and study quality were reported in few publications, and the median study met only one of a possible five study quality checklist items (IQR 0–2). Random allocation to group was reported by 67 of 427 publications (16%), blinded assessment of outcome by 95 (22%), a statement of potential conflict of interest by 54 (13%), compliance with animal welfare regulations by 239 (56%); and no publication reported a sample size calculation. The lack of sample size calculation was accompanied by small group sizes; for neurobehavioural outcomes, the median group size was 7 (IQR 7–14) in the control group and 9 (IQR 6–12) in the treatment group; and for pathological outcomes, 7 (IQR 5–9) and 7 (IQR 5–10), respectively.

Overall, stratifying pathological outcomes by aggregate study quality accounted for a significant proportion of the observed heterogeneity (χ^2^ = 142, 725 observations, p < 0.03, Figure 4, Table S2); however, there was no clear relationship between study quality and effect size. Similarly, stratifying neurobehavioral summary data accounted for a significant proportion of the observed heterogeneity but a clear relationship could not be defined (χ^2^ = 10.7, p < 0.03, Figure 4, 259 observations, Table S2).

**Figure 5:**
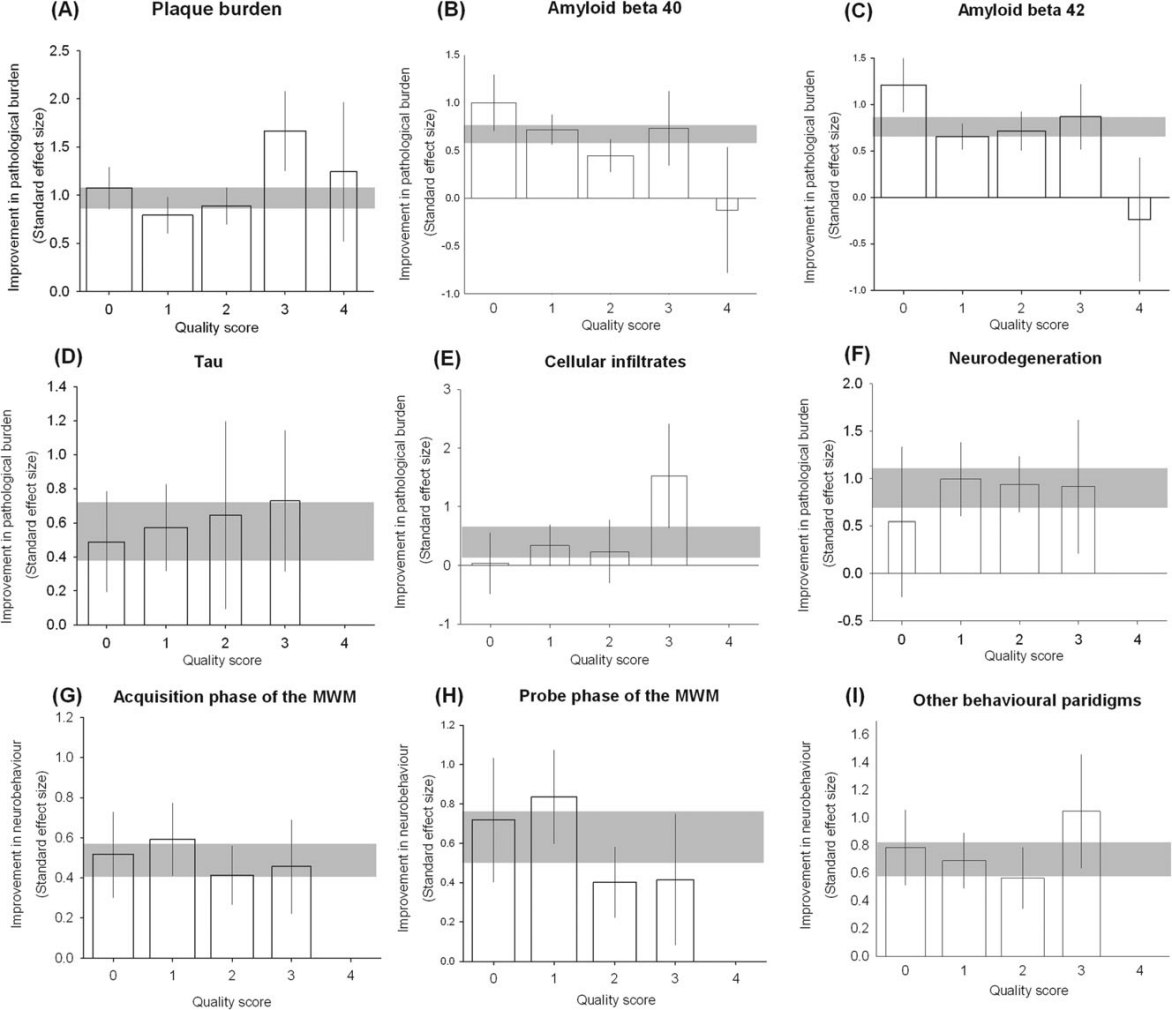
Impact of study quality items across all reported outcomes. We stratified outcomes to identify whether there was an association between effect size and overall study quality across (**A**) plaque burden, (**B**) amyloid beta 40, (**C**) amyloid beta 42, (**D**) tau, (**E**) cellular infiltrates (**F**) neurodegeneration, (**G**) acquisition phase of the Morris water maze, (**H**) probe phase of the Morris water maze and (**I**) non‐Morris water maze‐based behavioural paradigms. For each outcome, the horizontal grey bar represents 95% confidence interval (CI) of the global estimate, vertical error bars represent 95% CI of summary estimates and bar width represents the log of the number of animals.

We identified four individual pathological outcomes where stratification by aggregate study quality explained a significant proportion of the observed heterogeneity. In studies reporting a change in plaque burden, amyloid beta 42, tau, and cell infiltrates, the number of checklist items scored accounted for a significant proportion of between study heterogeneity (χ^2^ = 110, 23.3, 13.4 and 129, respectively, df = 4, p < 0.009, Figure 5, Table S2).

Overall, reported efficacy was 3.4% higher in non‐randomized studies (relative difference, 95% CI 1.8–5.0%, see Figure S2). There was an apparent difference between pathological outcomes (9.0% lower, 95% CI ‐11.8 to ‐6.0%) and behavioural outcomes (28% higher, 95% CI 26.1–30.7%), but partitioning by the type of outcome did not explain a significant proportion of the observed heterogeneity (p = 0.053). Overall, reported efficacy was 5.8% higher in non‐blinded studies (95% CI 2.8–8.8%; Figure S3); for pathological outcomes efficacy was 2.6% higher (95% CI −1.5% to 6.7%) and for behavioural outcomes efficacy was 13.1% higher (6.6–19.6%); partitioning heterogeneity by the type of outcome did explain a significant proportion of the observed heterogeneity (p = 0.014).

### PUBLICATION BIAS

**Figure 6:**
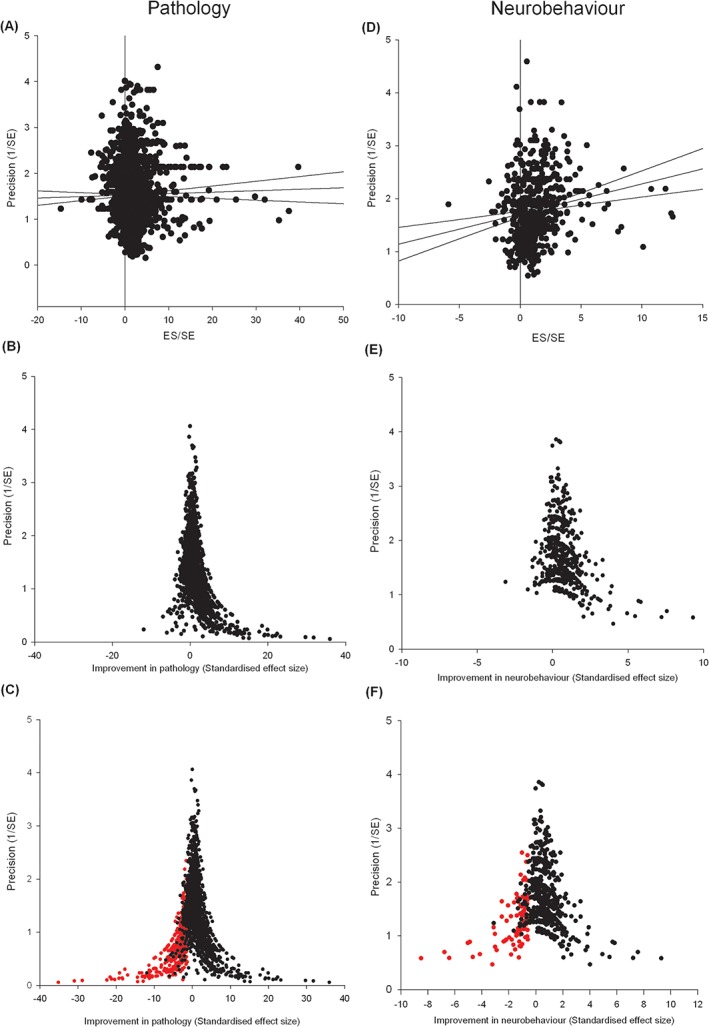
Publication bias assessment across pathological and behavioural outcomes: summary data for pathological and neurobehavioral outcomes. Pathological outcomes are assessed by (**A**) Egger regression, (**B**) funnel plot asymmetry and (**C**) Trim and fill techniques (missing studies shown in red). Likewise neurobehavioural data are assessed through Egger regression (**D**), funnel plot asymmetry and (**E**) Trim and fill techniques (**F**).

To investigate publication bias, we assessed all pathological outcomes using Egger regression, which did suggest missing studies (Figure 6A, see Table 2 for overview). Funnel plot asymmetry was also consistent with publication bias (Figure 6B). Trim and fill suggested that observed efficacy of 0.749 SD (95% CI 0.702–0.796, 2,517 experiments) was reduced to 0.419 SD (0.367–0.470) after the inclusion of 483 imputed missing studies (Figure 6C). For neurobehavioral outcomes, we again found evidence of publication bias with both Egger regression and funnel plot asymmetry (Figure 6D and E). Trim and fill suggested a baseline efficacy of 0.600 (0.537‐0.664), 561 experiments) which was reduced to 0.400 (0.329 to 0.471) after the inclusion of 98 imputed missing studies (Figure 6F, Table 2).

**Table 2:** Publication bias analyses: summary table of assessing pathological outcomes for the presence of publication bias through Egger regression, Funnel plot asymmetry and Trim and fill techniques. Where Trim and fill identified publication bias, both the unadjusted and adjusted estimates of efficacy are given alongside the percentage of experiments which are hypothesized missing.

Outcome measure	Publication bias identified from:		“Trim and Fill” global estimate (standard deviations [95% confidence interval] and N)		Number missing	Global estimate	
	Egger	Funnel plotting	Unadjusted	Adjusted	(%)	Absolute change (SD)	Relative change (%)
Plaque burden	Y	Y	0.999	0.61			
(antibody stained)			(0.905–1.093)	(0.508–0.712)	154	0.389	63.8
			632	786	(−19.6)		
Amyloid beta 40	Y	Y	0.635	0.321			
			(0.547–0.724)	(0.221–0.421)	124	0.314	97.8
			625	749	(−16.6)		
Amyloid beta 42	Y	Y	0.706	0.351			
			(0.616–0.796)	(0.250–0.452)	136	0.355	101.1
			632	768	(−17.7)		
NFT	Y	Y	0.533	0.285			
			(0.400–0.666)	(0.141–0.430)	43	0.248	87
			273	316	(−13.6)		
Cell infiltrates	Y	N	0.561	0.561			
			(0.367–0.755)	(0.367–0.755)	0	0	0%
			222	222	0		
Neurodegeneration	Y	Y	0.962	0.764			
			(0.784–1.140)	(0.566–0.961)	17	0.198	25.9
			133	150	(−11.3)		
Pathology	Y	Y	0.749	0.419		0.33	78.8
			(0.702–0.796)	(0.367–0.470)	483		
			2,517	3000	(−16.1)		
Acquisition phase of MWM	Y	Y	0.486	0.349			
			(0.402–0.570)	(0.260–0.438)	32	0.137	39.3
			164	196	(−16.3)		
Probe phase of MWM	Y	Y	0.623	0.4		0.223	
			(0.503–0.744)	(0.262–0.539)	32		55.8
			212	244	(−13)		
Fear conditioning	Y	Y	0.693	0.503			
			(0.495–0.890)	(0.297–0.709)	10	0.19	37.8
			60	70	(−14.3)		
Radial arm water maze	Y	Y	0.804	0.507			
			(0.585–1.024)	(0.279–0.735)	16	0.297	58.6
			53	69	(−23.1)		
T and Y maze	Y	Y	0.382	0.299			
			(0.166–0.597	(0.068–0.530)	3	0.083	27.8
			42	45	(−6.7)		
Novel object recognition task	Y	Y	0.904	0.629			
			(0.618–1.190)	(0.300–0.959)	6	0.275	43.7
			30	36	(−16.7)		
Neurobehaviour	Y	Y	0.6	0.4	98	0.2	50
			(0.537–0.664)	(0.329–0.471)	(−14.9)		
			561	659			

To ensure our findings of publication bias were not because of differences between outcomes, transgenic model groups or brain regions (wherever appropriate), we performed sensitivity analysis in strata of specific outcomes measures, transgenic model groups and brain areas and found persisting evidence of publication bias in all analyses save trim and fill analysis of cellular infiltrates outcomes.

## Discussion

In this systematic review, we identified 427 publications reporting data from 55 different transgenic mouse models testing 357 separate intervention strategies. Measures to avoid bias such as blinding and randomization were rarely reported, and the absence of sample size calculations was accompanied by low group sizes across all reported data. Only 16 of the 357 interventions were tested in more than 4 publications and trim and fill analysis suggested that 1 in 7 pathological and neurobehavioural remain unpublished. Collectively, these findings provide further insight into plausible reasons for translational failure.

### LIMITATIONS

There are a number of limitations to this work. For the analyses conducted, we only include data in the public domain, and we have shown evidence of a significant publication bias. However, this is likely to be less distorting that is the case for narrative reviews. Secondly, this is in essence an observational study, and the differences observed do not confirm causal relationships; our findings should be considered hypothesis generating only.

As with individual studies using transgenic animals some findings may be because of specific mutations, zygosity, promoter or background strain, but the relative consistency of findings between different mouse strains and different laboratories suggest that it is possible to generalize from findings beyond the specific details of individual experiments. Further, for the majority of our neurobehavioural data, our restriction to limit this to the last time point available would not have detected a transient improvement in function; however, we took the view that the most clinically relevant outcome was the longest term outcome available.

We have assessed the risks of bias on the basis of study reports, and it may be that measures to reduce the risk of bias were taken but not reported. However, since the assessment of risk of bias is a central component of critical reading of the scientific literature, publications which do not report measures to reduce the risk of bias should, in our view, be considered to be at higher risk of bias than those which do report such measures. Such issues are widespread: recent evidence has highlighted that measures to avoid bias in published literature are independent of the journal of publication.^11^

The search was conducted in 2009, and so cannot be taken to be representative of current AD modelling in either the prevalence or impact of biases because of study design or publication bias. Indeed, there is some evidence^11^ of improvement in reporting across the life sciences, perhaps because of, for example, the ARRIVE and Landis guidelines. However, at worse this gives us a baseline against which to measure such improvements, and we provide further motivation, if any is needed, for continuing efforts to improve the reporting, and perhaps the conduct, or *in vivo* research in AD as elsewhere.

Finally, selecting primary outcome measures on the basis of their prevalence in the literature could encourage analysis on the basis of those outcomes favoured by the community, which might not be the most clinically relevant outcomes. However, in the absence of consensus to the most relevant outcomes in animals, we believe that our approach is reasonable.

### INTERNAL VALIDITY

Notwithstanding these limitations, we have shown that the reporting of fundamental study quality items^12^ was relatively low. Overall, both randomization and blinding were associated with smaller effect sizes. Behavioural outcome measures appeared to be more susceptible to these biases than did pathological outcome measures, and for the blinded assessment of outcome this reached statistical significance. Interestingly, for pathological outcomes, randomization was associated with larger estimates of efficacy; we cannot explain this observation. It is also interesting to note that the impact of blinding and randomization appears to be smaller than in animal models of other diseases.^13^

The reporting of a power calculation is important both to reassure the reader that group size has not been inflated while the experiment was in progress and that the study was adequately powered to confirm or refute a biologically important effect. We and others have presented evidence that animal studies modelling multiple sclerosis and AD^5^, ^14^ are usually underpowered, and against this background it is troubling that not one of the 427 studies reported a sample size calculation, and that the sample sizes were generally small.

### EXTERNAL VALIDITY

A crucial issue in the modelling of AD is the stage of disease at which treatment is initiated;^2^, ^14^ our data suggest that most interventions are initiated relatively early in the mouse lifespan, being a median of 168 days (IQR 84–308) for pathological outcomes and 140 days (IQR 84–280) for neurobehavioural outcomes.

We found remarkable variation in experimental approaches used and outcome measures reported. This may be a function of reporting bias, with only those outcomes giving statistically significant findings or those consistent with the hypothesis under investigation being reported. For the probe phase of the MWM (where we identified 12 different outcome measures) previous work has suggested that the most sensitive outcome measure is the mean proximity to the former platform location^15^ but this was not commonly used. Pathological outcomes were reported twice as frequently as neurobehavioural outcomes although it should be noted that this may be partly explained by limitations of the animal models themselves. Of those reporting pathological outcomes only 1 in 10 reported changes in tau or neurodegeneration—both considered important features of human disease. Further insight into trial design may be provided if we can extend this work to identify those pathological outcomes which correlate best with improvements in neurobehavioural outcome in animals, as these may give a better indication of potential efficacy in human studies.

### PUBLICATION BIAS

Our publication bias analyses suggested that there is a large body of missing neutral or negative studies, and accounting for these led to substantial downward revisions in our estimates of pathological and neurobehavioural efficacy. This is of concern because it suggests that clinical trials may be based on incomplete data, and that there may be unnecessary repetition of animal experiments at the preclinical trial stage.

## Conclusions

There is a substantial literature describing the efficacy of various interventions in transgenic models of AD. We found that preclinical studies are characteristically diverse and few studies report fundamental study quality items (e.g. blinding, randomization); risk of bias does indeed impact on the observed efficacy; and an extensive publication bias is present across the transgenic mouse model literature. As a scientific community, collectively addressing these components of research design would decrease the risk of overstating efficacy from experiments conducted in animal models of AD. Collectively, these findings confirm that we cannot take evidence from preclinical trials at face value, and further demonstrate the utility of systematic review and meta‐analysis.

## Conflict of Interest

The authors have no conflicts of interest to declare.

## Supporting information

Figure S1. Construct validity issues in the use of the open field test: for outcomes from the open field test, the presence of a transgene was associated with both an increase and decrease in ambulation. Data were “normalised” whereby control transgenic outcomes equated to 100%. Error bars represent the standard deviation of each estimate and colours represents transgenic model group used.
Figure S2. Risk of bias for randomization: overstatement of efficacy in studies which did not report random allocation to group. Nft, neurofibrillary tangles; inf, cellular infiltrates; Neurod, neurodegeneration.
Figure S3. Risk of bias for blinding: overstatement of efficacy in studies which did not report blinding their assessment of outcome. Nft, neurofibrillary tangles; inf, cellular infiltrates; Neurod, neurodegeneration.

Table S1. Probe phase of the Morris water maze. Twelve principal methods were used to assess Morris water maze probe performance. Within these assessments studies varied by whether they; trained the mice to criterion, preformed multiple trials, assessed probe performance less than (<24) or greater than (>24) 24 hours after training, and the total number of seconds.
Table S2. Impact on overall quality on observed effect size: we stratified outcomes to identify whether there was an association between effect size and overall study quality score. For each outcome, summary estimates are provided for effect size, 95% confidence limits. Results are significant where stratification accounted for a significant proportion of the observed heterogeneity (*).
Table S3. Impact of blinding and randomization: we stratified outcomes to identify whether there was an association between effect size and blinding or randomization. For each outcome, summary estimates are provided for effect size, 95% confidence interval. Results are significant where stratification accounted for a significant proportion of the observed heterogeneity.
