# Supplementary material for: From a mouse: systematic analysis reveals limitations of experiments testing interventions in Alzheimer's disease mouse models
Source: Evid Based Preclin Med. 2016 Jul 22;3(1):e00015. doi: 10.1002/ebm2.15 (PMC5703440; doi:10.1002/ebm2.15)
Supplement: Supplementary file 1 — Figure S1. Construct validity issues in the use of the open field test: for outcomes from the open field test, the presence of a transgene was associated with both an increase and decrease in ambulation. Data were “normalised” whereby control transgenic outcomes equated to 100%. Error bars represent the standard deviation of each estimate and colours represents transgenic model group used. Figure S2. Risk of bias for randomization: overstatement of efficacy in studies which did not report random allocation to group. Nft, neurofibrillary tangles; inf, cellular infiltrates; Neurod, neurodegeneration. Figure S3. Risk of bias for blinding: overstatement of efficacy in studies which did not report blinding their assessment of outcome. Nft, neurofibrillary tangles; inf, cellular infiltrates; Neurod, neurodegeneration. [file EBM2-3-12-s001.docx]

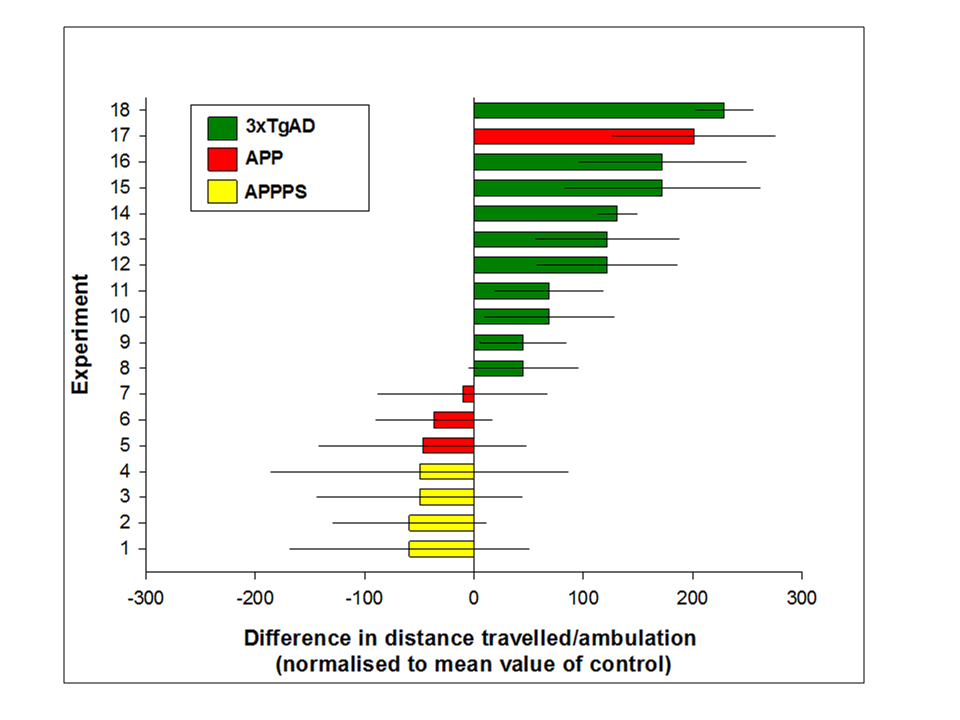


**Supplementary Figure 1: Construct validity issues in the use of the open field test:** For outcomes from the open field test, the presence of a transgene was associated with both an increase and decrease in ambulation. Data were ‘normalised’ whereby control transgenic outcomes equated to 100%. Error bars represent the standard deviation of each estimate and colours represents transgenic model group used.


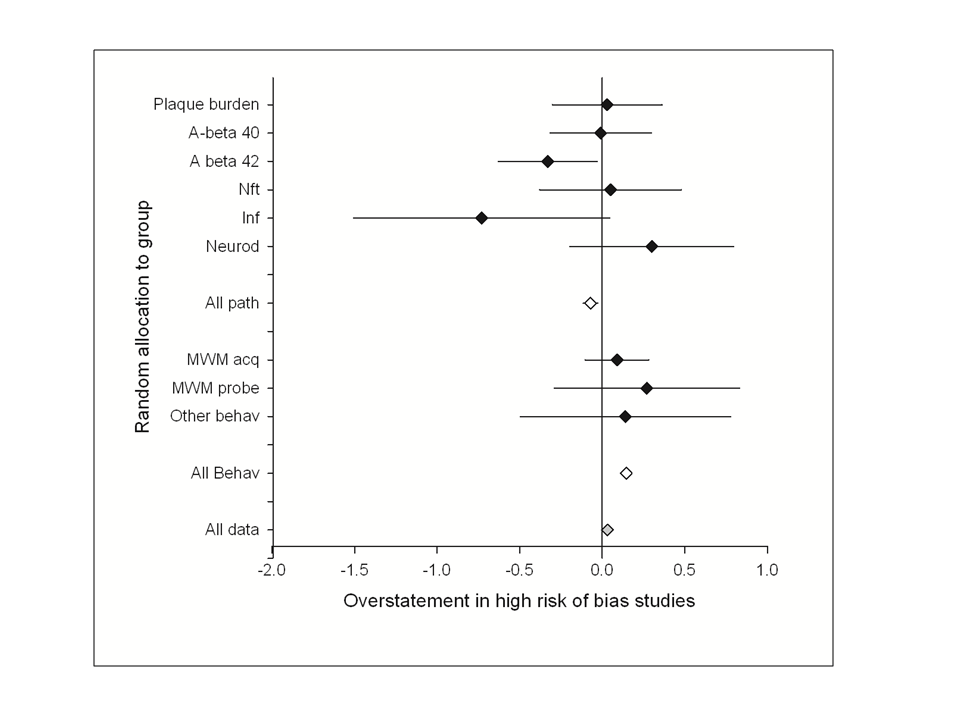


**Supplementary Figure 2: Risk of Bias for randomisation:** Overstatement of efficacy in studies which did not report random allocation to group. Nft: neurofibrillary tangles, inf: cellular infiltrates, Neurod: Neurodegeneration.


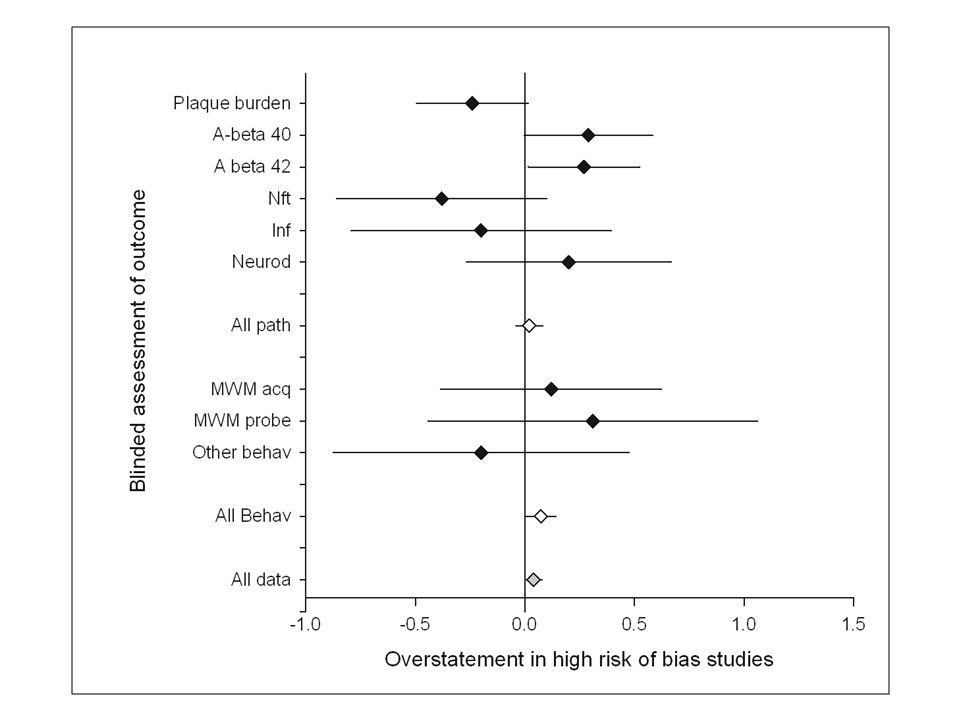


**Supplementary Figure 3: Risk of Bias for blinding:** Overstatement of efficacy in studies which did not report blinding their assessment of outcome. Nft: neurofibrillary tangles, inf: cellular infiltrates, Neurod: Neurodegeneration.
