# Supplementary material for: From a mouse: systematic analysis reveals limitations of experiments testing interventions in Alzheimer's disease mouse models
Source: Evid Based Preclin Med. 2016 Jul 22;3(1):e00015. doi: 10.1002/ebm2.15 (PMC5703440; doi:10.1002/ebm2.15)
Supplement: Supplementary file 2 — Table S1. Probe phase of the Morris water maze. Twelve principal methods were used to assess Morris water maze probe performance. Within these assessments studies varied by whether they; trained the mice to criterion, preformed multiple trials, assessed probe performance less than (<24) or greater than (>24) 24 hours after training, and the total number of seconds. Table S2. Impact on overall quality on observed effect size: we stratified outcomes to identify whether there was an association between effect size and overall study quality score. For each outcome, summary estimates are provided for effect size, 95% confidence limits. Results are significant where stratification accounted for a significant proportion of the observed heterogeneity (*). Table S3. Impact of blinding and randomization: we stratified outcomes to identify whether there was an association between effect size and blinding or randomization. For each outcome, summary estimates are provided for effect size, 95% confidence interval. Results are significant where stratification accounted for a significant proportion of the observed heterogeneity. [file EBM2-3-12-s002.docx]

**Supplementary Tables**

| **Method of probe assessment** | **Trials to criterion** | **Multiple probe trials** | **<24 hours** | **>24 hours** | **Total number of seconds** | **Number of publications** |
| --- | --- | --- | --- | --- | --- | --- |
| **Time in target quadrant** | no | no | no | yes | 31 to 60 | 13 |
|  |  |  |  |  | 61 to 120 | 3 |
|  |  |  |  |  | unknown | 2 |
|  |  |  | yes | no | 31 to 60 | 6 |
|  |  |  |  |  | 61 to 120 | 2 |
|  |  |  | unknown | unknown | 31 to 60 | 2 |
|  |  |  |  |  | 61 to 120 | 1 |
|  |  |  |  |  | unknown | 2 |
|  |  |  | Yes * | no | 31 to 60 | 1 |
|  |  | yes | no | yes | 31 to 60 | 1 |
|  |  |  | yes | no | 31 to 60 | 2 |
|  |  |  |  |  | unknown | 1 |
|  |  |  |  | yes | 31 to 60 | 1 |
|  |  |  | no | yes | 31 to 60 | 1 |
|  |  |  | Yes^*^ | no | 0 to 30 | 1 |
|  |  |  |  |  | 31 to 60 | 2 |
|  | yes | no | no | yes | 31 to 60 | 2 |
|  |  | yes | yes | yes | 31 to 60 | 1 |
| **Time in target quadrant Total** |  |  |  |  |  | **44** |
| **Number of platform crosses** | no | no | no | yes | 31 to 60 | 7 |
|  |  |  |  |  | 61 to 120 | 3 |
|  |  |  |  |  | unknown | 1 |
|  |  |  | yes | no | 31 to 60 | 2 |
|  |  |  |  |  | 61 to 120 | 1 |
|  |  |  |  |  | unknown | 1 |
|  |  |  | unknown | unknown | 61 to 120 | 1 |
|  |  | yes | Yes * | no | 31 to 60 | 1 |
|  |  |  |  | yes | 31 to 60 | 1 |
|  | yes | yes | yes | yes | 31 to 60 | 2 |
|  |  |  |  |  | unknown | 2 |
| **Number of platform crosses Total** |  |  |  |  |  | **22** |
| **Speed** | no | no | no | yes | 31 to 60 | 2 |
|  |  |  |  |  | 61 to 120 | 1 |
|  |  |  |  |  | unknown | 1 |
|  |  |  | yes | no | 31 to 60 | 1 |
|  |  | yes | no | yes | 31 to 60 | 1 |
|  |  |  | yes | no | 31 to 60 | 2 |
|  |  |  | Yes * | no | 0 to 30 | 1 |
|  |  |  |  |  | 31 to 60 | 1 |
| **Speed Total** |  |  |  |  |  | **10** |
| **Latency to cross platform** | no | no | no | yes | 31 to 60 | 1 |
|  |  | yes | no | yes | 31 to 60 | 1 |
|  |  |  | yes | no | 31 to 60 | 1 |
|  | yes | yes | yes | yes | 31 to 60 | 4 |
|  |  |  |  |  | unknown | 2 |
| **Latency to cross platform Total** |  |  |  |  |  | **9** |
| **Distance travelled to platform^1^** | no | no | no | yes | 31 to 60 | 1 |
|  |  |  |  |  | 61 to 120 | 2 |
|  |  | yes | no | yes | 31 to 60 | 1 |
|  |  |  | yes | no | 31 to 60 | 1 |
| **Distance travelled to platform Total** |  |  |  |  |  | **5** |
| **Number of entries to target quadrant** | no | no | no | yes | unknown | 1 |
|  |  |  | unknown | unknown | unknown | 1 |
|  |  | yes | yes | no | unknown | 1 |
|  | yes | yes | yes | yes | 31 to 60 | 1 |
| **Number of entries to target quadrant Total** | |  |  |  |  | **4** |
| **Distance travelled in target quadrant** | no | no | no | yes | 31 to 60 | 1 |
|  |  |  | yes | no | 31 to 60 | 1 |
|  |  | yes | yes | yes | 31 to 60 | 1 |
| **Distance travelled in target quadrant Total** | |  |  |  |  | **3** |
| **Number of entries to target zone** | no | no | no | yes | 61 to 120 | 1 |
|  |  |  |  |  | unknown | 1 |
| **Number of entries to target zone Total** | |  |  |  |  | **2** |
| **Average distance to platform** | yes | no | no | yes | 31 to 60 | 2 |
| **Average distance to platform Total** |  |  |  |  |  | **2** |
| **Time at platform** | no | no | no | yes | 61 to 120 | 1 |
| **Time at platform Total** |  |  |  |  |  | **1** |
| **Time in target zone** | no | no | no | yes | 61 to 120 | 1 |
| **Time in target zone Total** |  |  |  |  |  | **1** |
| **Search Ratio** | yes | no | no | yes | 31 to 60 | 1 |
| **Search Ratio Total** |  |  |  |  |  | **1** |

**Supplementary Table 1 (previous page):** Probe phase of the Morris water maze Summary table of assessing pathological outcomes for the presence of publication bias through Egger regression, Funnel plot asymmetry and Trim and fill techniques. Where Trim and fill identified publication bias both the unadjusted and adjusted estimates of efficacy are given alongside the percentage of experiments which are hypothesised missing.

| **Outcome measure** | **Aggregate Study Quality** | | | | | **Combined** |
| --- | --- | --- | --- | --- | --- | --- |
|  | **0** | **1** | **2** | **3** | **4** |  |
| **Plaque burden*** | 1.07 SD | 0.79 SD | 0.89 SD | 1.67 SD | 1.24 SD | 0.98 SD |
| **(antibody stained)** | (0.85 to 1.29) | (0.60 to 0.98) | (0.7 to 1.08) | (1.25 to 2.08) | (0.52 to 1.97) | (0.87 to 1.1) |
|  | **91** | **151** | **99** | **35** | **2** | **378 (χ 2= 110)** |
| **Amyloid beta 40** | 1 SD | 0.72 SD | 0.45 SD | 0.73 SD | -0.13 SD | 0.68 SD |
|  | (0.7 to 1.29) | (0.56 to 0.87) | (0.27 to 0.62) | (0.34 to 1.12) | (-0.78 to 0.53) | (0.57 to 0.79) |
|  | **68** | **211** | **81** | **27** | **1** | **388 (χ 2= 9.27)** |
| **Amyloid beta 42*** | 1.21 SD | 0.66 SD | 0.72 SD | 0.87 SD | -0.24 SD | 0.78 SD |
|  | (0.92 to 1.5) | (0.51 to 0.8) | (0.51 to 0.93) | (0.51 to 1.22) | (-0.9 to 0.43) | (0.67 to 0.88) |
|  | **70** | **201** | **81** | **36** | **1** | **389 (χ 2= 23.3)** |
| **NFT** | 0.49 SD | 0.57 SD | 0.64 SD | 0.73 SD |  | 0.55 SD |
|  | (0.19 to 0.78) | (0.32 to 0.83) | (0.09 to 1.2) | (0.31 to 1.14) | No studies | (0.38 to 0.72) |
|  | **15** | **52** | **6** | **11** |  | **84 (χ 2= 13.4)** |
| **Cell infiltrates*** | 0.04 SD | 0.34 SD | 0.23 SD | 1.53 SD |  | 0.4 SD |
|  | (-0.48 to 0.56) | (-0.01 to 0.7) | (-0.3 to 0.77) | (0.64 to 2.41) | No studies | (0.13 to 0.68) |
|  | **20** | **30** | **27** | **12** |  | **89 (χ 2= 129)** |
| **Neurodegeneration** | 0.54 SD | 0.99 SD | 0.94 SD | 0.91 SD |  | 0.91 SD |
|  | (-0.25 to 1.33) | (0.6 to 1.38) | (0.64 to 1.23) | (0.21 to 1.62) | No studies | (0.69 to 1.12) |
|  | **7** | **24** | **25** | **8** |  | **64 (χ 2= 2.8)** |
|  | 0.96 SD | 0.72 SD | 0.65 SD | 1.09 SD | 0.66 SD | 0.78 SD |
| **Total Pathology** | (0.8 to 1.13) | (0.62 to 0.82) | (0.52 to 0.78) | (0.82 to 1.37) | (0.35 to 0.97) | (0.71 to 0.85) |
|  | **146** | **348** | **164** | **65** | **2** | **725 (χ 2=142)** |
| **Acquisition phase of MWM** | 0.52 SD | 0.59 SD | 0.41 SD | 0.46 SD |  | 0.49 SD |
|  | (0.3 to 0.73) | (0.41 to 0.77) | (0.26 to 0.56) | (0.22 to 0.69) | No studies | (0.40 to 0.58) |
|  | 25 | 50 | 29 | 26 |  | 130 **(χ 2= 1.80)** |
| **Probe phase of MWM*** | 0.72 SD | 0.84 SD | 0.4 SD | 0.41 SD |  | 0.63 SD |
|  | (0.4 to 1.03) | (0.6 to 1.07) | (0.22 to 0.58) | (0.08 to 0.75) | No studies | (0.5 to 0.76) |
|  | **24** | **47** | **26** | **16** |  | **113 (χ 2= 13.8)** |
| **Other NBS studies** | 0.78 SD | 0.69 SD | 0.56 SD | 1.05 SD |  | 0.72 SD |
|  | (0.51 to 1.05) | (0.49 to 0.89) | (0.34 to 0.79) | (0.63 to 1.46) | No studies | (0.59 to 0.84) |
|  | **28** | **39** | **38** | **18** |  | 123 (χ 2= 6.4) |
| **Total Behaviour** | 0.66 SD | 0.70 SD | 0.45 SD | 0.64 SD |  | 0.61 SD |
|  | (0.49 to 0.82) | (0.57 to 0.84) | (0.33 to 0.58) | (0.44 to 0.85) | No studies | (0.54 to 0.69) |
|  | **62** | **86** | **67** | **44** |  | **259 (χ 2= 10.7)** |

**Supplementary Table 2: Impact on overall quality on observed effect size** We stratified outcomes to identify whether there was an association between effect size and overall study quality score. For each outcome summary estimates are provided for effect size, 95% confidence limits. Results are significant where stratification accounted for a significant proportion of the observed heterogeneity(*).

| **Outcome** | **Study quality measure** | |
| --- | --- | --- |
|  | **Blinded (Difference in effect size and 95% CI)** | **Randomised**  **(Difference in effect size and 95% CI)** |
| **Plaque burden*** | -0.24  (-0.5 to 0.02) | 0.03  (-0.3 to 0.36) |
| **Amyloid beta 40** | 0.29  (0 to 0.58) | -0.01  (-0.32 to 0.3) |
| **Amyloid beta 42*** | 0.27  (0.01 to 0.53) | -0.33 (-0.63 to -0.03) |
| **NFT** | -0.38  (-0.86 to 0.1) | 0.05  (-0.38 to 0.48) |
| **Cell infiltrates*** | -0.2  (-0.8 to 0.4) | -0.73  (-1.51 to 0.05) |
| **Neurodegeneration** | 0.2  (-0.27 to 0.67) | 0.3  (-0.2 to 0.8) |
| **Total Pathology** | 0.02  (-0.01 to 0.05) | -0.07  (-0.09 to -0.05) |
| **Acquisition phase of MWM** | 0.12  (-0.15 to 0.39) | 0.09  (-0.1 to 0.29) |
| **Probe phase of MWM*** | 0.31  (-0.07 to 0.69) | **0.27**  **(-0.02 to 0.56)** |
| **Other NBS studies** | -0.2  (-0.54 to 0.14) | 0.14  (-0.19 to 0.47) |
| **Total Behaviour** | 0.07  (0.04 to 0.11) | 0.14  (0.13 to 0.16) |
| **All outcomes** | **0.04**  **(0.02 to 0.06)** | **0.03**  **(0.02 to 0.04)** |

**Supplementary Table 3: Impact of blinding and randomisation:** We stratified outcomes to identify whether there was an association between effect size and blinding or randomisation. For each outcome summary estimates are provided for effect size, 95% confidence limits. Results are significant where stratification accounted for a significant proportion of the observed heterogeneity.
